# Supplementary material for: Hemodynamic and Clinical Profiles of Pulmonary Arterial Hypertension Patients with GDF2 and BMPR2 Variants
Source: Int J Mol Sci. 2024 Feb 27;25(5):2734. doi: 10.3390/ijms25052734 (PMC10931624; doi:10.3390/ijms25052734)
Supplement: Supplementary file 1 [file ijms-25-02734-s001.zip › ijms-2785520-supplementary.pdf]

SUPPLEMENTAL

Table S1. Details of genetic variants on *EIF2AK4* gene

No.	ID	Gender	Gene	cDNA change	Amino acid change	Variant type	Genotype	PolyPhen2 /SIFT	ACMG 2015*	MAF in EAS	Ref
	A100540	M	*EIF2AK4*	c.4218G>T	p.Gln1406His	Missense	Het	B/T	LB	0.01177	
	A100551	M	*EIF2AK4*	c.4218G>T	p.Gln1406His	Missense	Het	B/T	LB	0.01177	
	A100559	F	*EIF2AK4*	c.4218G>T	p.Gln1406His	Missense	Het	B/T	LB	0.01177	
	A100641	F	*EIF2AK4*	c.4218G>T	p.Gln1406His	Missense	Het	B/T	LB	0.01177	
	A110137	F	*EIF2AK4*	c.4218G>T	p.Gln1406His	Missense	Het	B/T	LB	0.01177	

1. Reference sequences: *EIF2AK4 (NM_001013703.4)*
2. PolyPhen2: PD, Probably damaging; D, Damaging; B, Benign. SIFT: D, Deleterious, T, Tolerated.
3. PolyPhen2 and SIFT *in silico* prediction program were unable to analyze frameshift, nonsense, in frame deletion, gross deletion types and some variants with unknown reasons.
4. ACMG 2015: The American College of Medical Genetics and Genomics guidelines： VUS, variant of uncertain significance; LB, Likely Benign.
5. MAF in EAS: Minor allele frequency of East Asian in gnomAD exome databases.

Table S2. Female pulmonary arterial hypertension patients with or without bone morphogenetic protein receptor type 2 (*BMPR2*) genetic variants, clinical and hemodynamic presentations at initial diagnosis (N=51).

Characteristics		*BMPR2* variant carrier (N=5)	Non-*BMPR2*, non-*GDF2* variant (N=46)	*p*-value
				BMPR2 versus non-*BMPR2* variant
Female (N, %))		5 (100)	46 (100)	
Age of onset, years		43 ± 11	54 ± 20.2	0.277
Six-minute walking distance (m)		351 ± 165	322 ± 123	0.613
Mean pulmonary arterial pressure (mmHg)		66 ± 15	37 ± 14	<0.001
Pulmonary arterial wedge pressure (mmHg)		11 ± 3	14 ± 5	0.198
Pulmonary vascular resistance (woods)		22 ± 9	7 ± 5	<0.001
Peak tricuspid regurgitation peak gradient (mmHg)		64 ± 35	49 ± 31	0.330
Peak oxygen consumption (mL/min/kg)		12 ± 4	12 ± 4	0.948
ventilatory equivalents for carbon dioxide (VE/VCO2)		50 ± 18	38 ± 13	0.088
Right atrial pressure (mmHg)		14 ± 9	14 ± 12	0.994
Cardiac index (L/min/m^2)		1.6 ± 0.5	2.7 ± 1.1	0.021
Pulmonary artery saturation (%)		54 ± 13	65 ± 12	0.060
N-terminal prohormone of brain natriuretic peptide (ng/L)		4117 ± 5336	1875 ± 2897	0.139
World Health Organization functional class (N, %)	I	1 (20)	3 (6.5)	0.490
	II	1 (20)	11 (23.9)	
	III	3 (60)	30 (65.2)	
	IV	0 (0)	2 (4.3)	
Progression of symptoms	No	1 (20)	19 (41.3)	0.502

	Slow	4 (80)	21 (45.7)
	Rapid	0 (0)	6 (13.0)

† Data of continuous variables were expressed as mean ± SD.

† Changes of categorical variables were analyzed by Chi-square tests and were expressed by (N, %).

Table S3. Male Pulmonary arterial hypertension patients with or without Growth Differentiation Factor 2 (*GDF2*) genetic variants, clinical and hemodynamic presentations at initial diagnosis (N=18).

Characteristics		*GDF2* variant carrier (N=3)	Non-*BMPR2*, non-*GDF2* variant (N=15)	*p*-value
				GDF2 versus non-*GDF2* variant
Male		3 (100)	15 (100)	
Age of onset, years		25 ± 13	44 ± 19	0.109
Six-minute walking distance (m)		490 ± 101	319 ± 151	0.082
Mean pulmonary arterial pressure (mmHg)		66 ± 13	39 ± 12	0.002
Pulmonary arterial wedge pressure (mmHg)		11 ± 3	14 ± 2	0.138
Pulmonary vascular resistance (woods)		15 ± 12	6 ± 5	0.048
Peak tricuspid regurgitation peak gradient (mmHg)		58 ± 23	54 ± 30	0.827
Peak oxygen consumption (mL/min/kg)		15 ± 6	14 ± 5	0.722
ventilatory equivalents for carbon dioxide (VE/VCO2)		45 ± 4	36 ± 17	0.500
Right atrial pressure (mmHg)		11 ± 5	12 ± 4	0.683
Cardiac index (L/min/m^2)		2.6 ± 1.1	2.9 ± 1.0	0.734
Pulmonary artery saturation (%)		68 ± 10	71 ± 8	0.612
N-terminal prohormone of brain natriuretic peptide (ng/L)		228 ± 46	1396 ± 2324	0.410
World Health Organization functional class (N, %)	I	0 (0)	5 (33.3)	0.840
	II	2 (66.7)	8 (53.3)	
	III	1 (33.3)	2 (13.3)	
	IV	0 (0)	0 (3.3)	
Progression of symptoms	No	1 (33.3)	5 (38.5)	0.670

Slow	1 (33.3)	8 (61.5)
Rapid	1 (33.3)	2 (15.4)

† Data of continuous variables were expressed as mean ± SD.

† Changes of categorical variables were analyzed by Chi-square tests and were expressed by (N, %).

Table S4. Pulmonary arterial hypertension patients with or without *ATP13A3* genetic variants, clinical and hemodynamic presentations at initial diagnosis (N=63).

Characteristics		*ATP13A3* variant carriers (N=4)	Non-*ATP13A3*, variant carriers (N=59)*	*p*-value
Female (N, %))		3 (75)	45 (76.3)	
Age of onset, years		48.0 ± 8.6	51.4 ± 20.4	0.746
Six-minute walking distance (m)		320 ± 167	319 ± 122	0.982
Mean pulmonary arterial pressure (mmHg)		52 ± 7	38 ± 13	0.043
Pulmonary arterial wedge pressure (mmHg)		12 ± 2	14 ± 5	0.296
Pulmonary vascular resistance (woods)		15 ± 14	7 ± 5	0.353
Peak tricuspid regurgitation peak gradient (mmHg)		45 ± 29	51 ± 30	0.687
Peak oxygen consumption (mL/min/kg)		11 ± 6	12 ± 4	0.528
ventilatory equivalents for carbon dioxide (VE/VCO2)		44 ± 19	38 ± 14	0.406
Right atrial pressure (mmHg)		13 ± 8	14 ± 10	0.881
Cardiac index (L/min/m^2)		1.8 ± 0.8	2.8 ± 1.1	0.078
Pulmonary artery saturation (%)		57 ± 12	67 ± 12	0.116
N-terminal prohormone of brain natriuretic peptide (ng/L)		4254 ± 5876	1782 ± 2806	0.120
World Health Organization functional class (N, %)	I	0 (0)	3 (5.1)	0.656
	II	0 (0)	16 (27.1)	
	III	3 (100)	38 (64.4)	
	IV	0 (0)	2 (3.4)	
Progression of symptoms	No	1 (25)	23 (40.0)	0.704
	Slow	2 (50)	29 (49.2)	

Rapid	1 (25)	7 (11.9)

† Data of continuous variables were expressed as mean ± SD.
† Changes of categorical variables were analyzed by Chi-square tests and were expressed by (N, %).
* Non-ATP13A3 carrier group also excluded patients carrying variants on *BMPR2* and *GDF2*.

Table S5 . Pulmonary arterial hypertension patients with or without *EIF2AK4* genetic variants, clinical and hemodynamic presentations at initial diagnosis (N=62).

Characteristics		*EIF2AK4* variant carriers (N=9)	Non-*EIF2AK4* variant carriers (N=53)*	*p*-value
Female (N, %))		3 (75)	45 (76.3)	
Age of onset, years		52.4 ± 23.6	50.4 ± 20.1	0.779
Six-minute walking distance (m)		334 ± 153	321 ± 117	0.767
Mean pulmonary arterial pressure (mmHg)		42 ± 12	38 ± 14	0.345
Pulmonary arterial wedge pressure (mmHg)		14 ± 6	14 ± 4	0.798
Pulmonary vascular resistance (woods)		6 ± 4	7 ± 5	0.470
Peak tricuspid regurgitation peak gradient (mmHg)		50 ± 43	50 ± 28	0.983
Peak oxygen consumption (mL/min/kg)		14 ± 4	12 ± 4	0.226
ventilatory equivalents for carbon dioxide (VE/VCO2)		36 ± 10	38 ± 15	0.744
Right atrial pressure (mmHg)		11 ± 5	14 ± 11	0.450
Cardiac index (L/min/m^2)		3.1 ± 1.3	2.7 ± 1.0	0.421
Pulmonary artery saturation (%)		66 ± 12	67 ± 11	0.951
N-terminal prohormone of brain natriuretic peptide (ng/L)		3526 ± 5117	1428 ± 2027	0.258
World Health Organization functional class (N, %)	I	0 (0)	3 (5.9)	0.417
	II	1 (11)	16 (31.4)	
	III	8 (89)	32 (62.7)	
	IV	0 (0)	2 (3.9)	
Progression of symptoms	No	3 (33.3)	21 (39.6)	0.897

	Slow	5 (55.6)	25 (47.2)
	Rapid	1 (11.1)	7 (13.2)

† Data of continuous variables were expressed as mean ± SD.

† Changes of categorical variables were analyzed by Chi-square tests and were expressed by (N, %).

* Non-*EIF2AK4* carriers group also excluded patients carrying variants on *BMPR2* and *GDF2*.

Supplemental Figure 1.

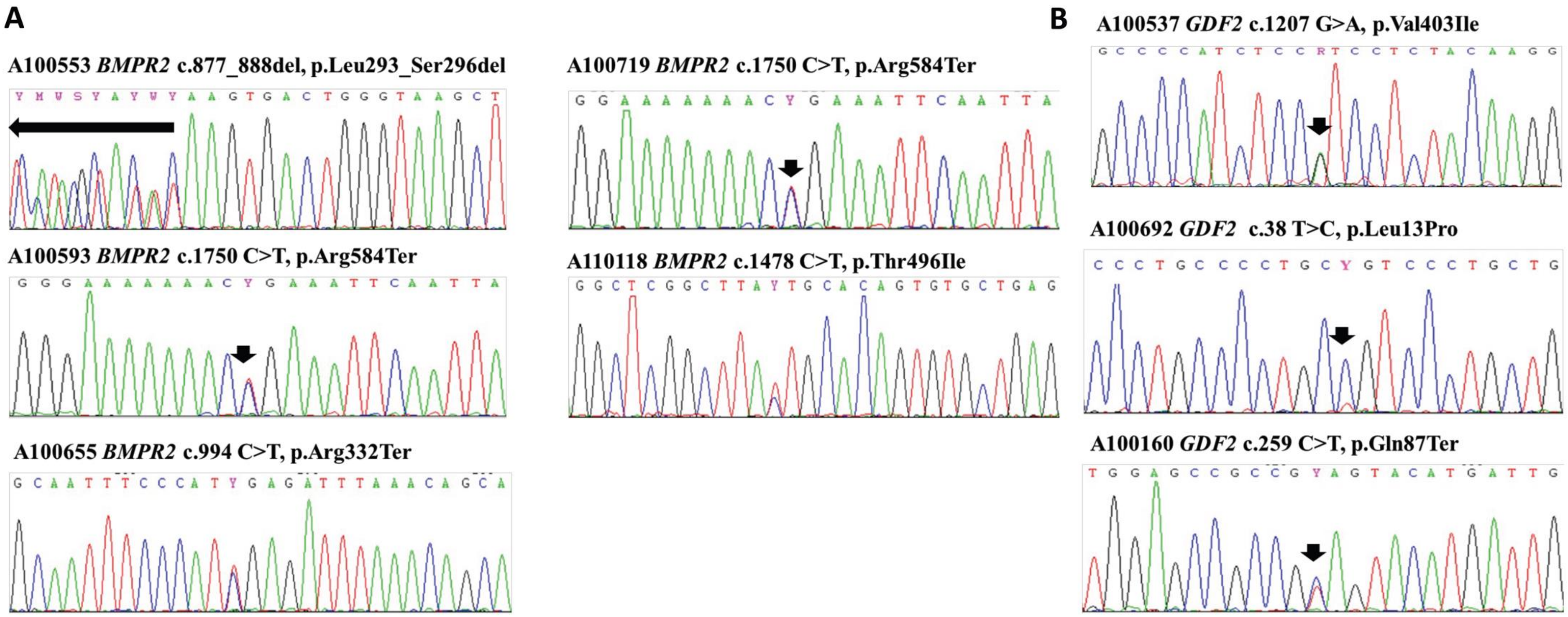

Supplement Figure S1. Sanger sequencing validation of variants on (A) BMPR2 and (B) GDF2. Five BMPR2 variants and three GDF2 variants from IPAH patients were confirmed by PCR-Sanger sequencing directly. Black arrows indicated exact variant. Y means C to T transition. R means A to G transition.
